# 8-Hy­droxy-2-methyl­quinolinium dibromido(2-methyl­quinolin-8-olato-κ^2^ *N*,*O*)zincate(II) methanol monosolvate

**DOI:** 10.1107/S1600536810036706

**Published:** 2010-09-18

**Authors:** Ezzatollah Najafi, Mostafa M. Amini, Seik Weng Ng

**Affiliations:** a Department of Chemistry, General Campus, Shahid Beheshti University, Tehran 1983963113, Iran; b Department of Chemistry, University of Malaya, 50603 Kuala Lumpur, Malaysia

## Abstract

The anion of the title salt, (C_10_H_10_NO)[ZnBr_2_(C_10_H_8_NO)]·CH_3_OH, has its metal atom *N*,*O*-chelated by the deprotonated 2-methyl-8-hy­droxy­quinoline ligand. The hy­droxy unit of the cation is a hydrogen-bond donor to the alkoxide O atom of the tetra­hedrally coordinated anion, whereas the ammonium cation is a hydrogen-bond donor to the methano­lic O atom. In the crystal, adjacent ion pairs and solvent mol­ecules are linked by a methanol–halogen O—H⋯Br hydrogen bond, generating a chain running along the *a* axis.

## Related literature

For the isostructural chloro analog, see: Sattarzadeh *et al.* (2009 ▶).
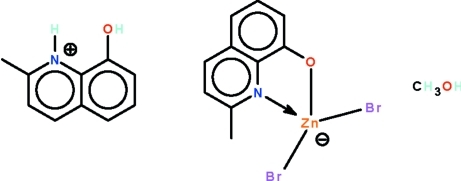


## Experimental

### 

#### Crystal data

(C_10_H_10_NO)[ZnBr_2_(C_10_H_8_NO)]·CH_4_O
*M* *_r_* = 575.60
Monoclinic, 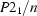

*a* = 9.9704 (8) Å
*b* = 13.9954 (11) Å
*c* = 15.8815 (12) Å
β = 105.815 (1)°
*V* = 2132.2 (3) Å^3^
*Z* = 4
Mo *K*α radiation
μ = 4.93 mm^−1^
*T* = 100 K
0.35 × 0.30 × 0.25 mm

#### Data collection

Bruker SMART APEX diffractometer
Absorption correction: multi-scan (*SADABS*; Sheldrick, 1996 ▶) *T*_min_ = 0.278, *T*_max_ = 0.372
19908 measured reflections
4889 independent reflections
4044 reflections with *I* > 2σ(*I*)
*R*_int_ = 0.040

#### Refinement

*R*[*F* ^2^ > 2σ(*F* ^2^)] = 0.025
*wR*(*F* ^2^) = 0.057
*S* = 1.03
4889 reflections
267 parameters
H-atom parameters constrained
Δρ_max_ = 0.49 e Å^−3^
Δρ_min_ = −0.37 e Å^−3^

### 

Data collection: *APEX2* (Bruker, 2009 ▶); cell refinement: *SAINT* (Bruker, 2009 ▶); data reduction: *SAINT*; program(s) used to solve structure: *SHELXS97* (Sheldrick, 2008 ▶); program(s) used to refine structure: *SHELXL97* (Sheldrick, 2008 ▶); molecular graphics: *X-SEED* (Barbour, 2001 ▶); software used to prepare material for publication: *publCIF* (Westrip, 2010 ▶).

## Supplementary Material

Crystal structure: contains datablocks global, I. DOI: 10.1107/S1600536810036706/bt5354sup1.cif

Structure factors: contains datablocks I. DOI: 10.1107/S1600536810036706/bt5354Isup2.hkl

Additional supplementary materials:  crystallographic information; 3D view; checkCIF report

## Figures and Tables

**Table 1:** Hydrogen-bond geometry (Å, °)

*D*—H⋯*A*	*D*—H	H⋯*A*	*D*⋯*A*	*D*—H⋯*A*
O2—H2⋯O1	0.84	1.71	2.546 (2)	172
O3—H3⋯Br1^i^	0.84	2.48	3.2941 (17)	163
N2—H2n⋯O3	0.86	1.91	2.739 (3)	162

Symmetry code: (i) 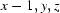.

**Fig. 1.**
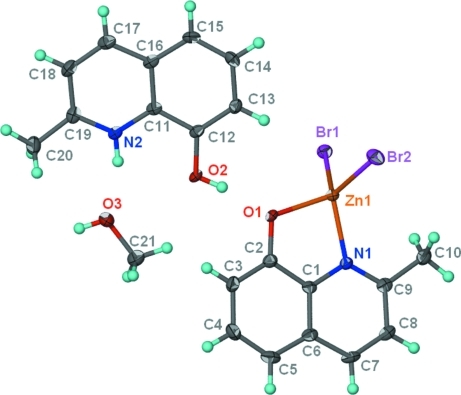
Anisotropic displacement ellipsoid plot (Barbour, 2001) of the title compound at the 70% probability level. Hydrogen atoms are drawn as spheres of arbitrary radius.

## supplementary crystallographic information

### Comment

An earlier study reported C_10_H_10_NO^+.^ZnCI_2_(C_10_H_8_NO)^.^CH_3_OH, which feature cations, tetrahedral anions and solvent molecules linked by N···O, O···O and O···Cl hydrogen bonds into a linear chain (Sattarzadeh *et al.*, 2009). The present bromide analog (Scheme I, Fig. 1) is isostructural, the two compounds displaying matching cell dimensions. The hydroxy unit of the cation is hydrogen-bond donor to the alkoxide O atom of the tetrahedrally coordinated anion whereas the ammonium unit is hydrogen-bond donor to the methanolic O atom. Adjacent ion-pairs and solvent molecules are linked by a *O*–H_methanol_···Br hydrogen bond to generate a linear chain running along the *a*-axis of the monoclinic unit cell.

### Experimental

Zinc bromide (0.10 g, 0.75 mmol) and 2-methyl-8-hydroxyquinoline (0.24 g, 1.5 mmol) were loaded into a convection tube; the tube was filled with dry methanol and kept at 333 K. Crystals were collected from the side arm after several days.

### Refinement

Hydrogen atoms were placed in calculated positions (C–H 0.95–0.98, N–H 0.86 and O–H 0.84 Å) and were included in the refinement in the riding model approximation, with *U*(H) set to 1.2–1.5*U*_eq_(C,*N*,*O*).

### Crystal data

**Table:**

(C_10_H_10_NO)[ZnBr_2_(C_10_H_8_NO)]·CH_4_O	*F*(000) = 1144
*M**_r_* = 575.60	*D*_x_ = 1.793 Mg m^−^^3^
Monoclinic, *P*2_1_/*n*	Mo *K*α radiation, λ = 0.71073 Å
Hall symbol: -P 2yn	Cell parameters from 6099 reflections
*a* = 9.9704 (8) Å	θ = 2.6–28.2°
*b* = 13.9954 (11) Å	µ = 4.93 mm^−^^1^
*c* = 15.8815 (12) Å	*T* = 100 K
β = 105.815 (1)°	Prism, yellow
*V* = 2132.2 (3) Å^3^	0.35 × 0.30 × 0.25 mm
*Z* = 4	

### Data collection

**Table:**

Bruker SMART APEX diffractometer	4889 independent reflections
Radiation source: fine-focus sealed tube	4044 reflections with *I* > 2σ(*I*)
graphite	*R*_int_ = 0.040
ω scans	θ_max_ = 27.5°, θ_min_ = 2.0°
Absorption correction: multi-scan (*SADABS*; Sheldrick, 1996)	*h* = −12→12
*T*_min_ = 0.278, *T*_max_ = 0.372	*k* = −18→18
19908 measured reflections	*l* = −19→20

### Refinement

**Table:**

Refinement on *F*^2^	Primary atom site location: structure-invariant direct methods
Least-squares matrix: full	Secondary atom site location: difference Fourier map
*R*[*F*^2^ > 2σ(*F*^2^)] = 0.025	Hydrogen site location: inferred from neighbouring sites
*wR*(*F*^2^) = 0.057	H-atom parameters constrained
*S* = 1.03	*w* = 1/[σ^2^(*F*_o_^2^) + (0.0224*P*)^2^ + 0.8543*P*] where *P* = (*F*_o_^2^ + 2*F*_c_^2^)/3
4889 reflections	(Δ/σ)_max_ = 0.001
267 parameters	Δρ_max_ = 0.49 e Å^−^^3^
0 restraints	Δρ_min_ = −0.37 e Å^−^^3^

### Fractional atomic coordinates and isotropic or equivalent isotropic displacement parameters (Å^2^)

**Table:**

	*x*	*y*	*z*	*U*_iso_*/*U*_eq_	
Br1	1.00294 (2)	0.228905 (17)	0.184698 (15)	0.01708 (6)	
Br2	1.12097 (3)	0.497149 (17)	0.227454 (16)	0.02018 (7)	
Zn1	0.99449 (3)	0.369708 (19)	0.266658 (17)	0.01403 (7)	
O1	0.79813 (16)	0.40171 (12)	0.25997 (10)	0.0166 (4)	
O2	0.58392 (16)	0.36051 (12)	0.13590 (10)	0.0155 (3)	
H2	0.6590	0.3718	0.1741	0.023*	
O3	0.30839 (18)	0.27619 (14)	0.15184 (11)	0.0255 (4)	
H3	0.2240	0.2660	0.1482	0.038*	
N1	1.01652 (19)	0.36488 (13)	0.39831 (12)	0.0120 (4)	
N2	0.35285 (19)	0.35026 (13)	0.00209 (12)	0.0129 (4)	
H2N	0.3540	0.3348	0.0547	0.015*	
C1	0.8930 (2)	0.38963 (15)	0.41453 (15)	0.0124 (5)	
C2	0.7784 (2)	0.40932 (16)	0.33955 (15)	0.0141 (5)	
C3	0.6537 (2)	0.43641 (17)	0.35416 (16)	0.0168 (5)	
H3a	0.5762	0.4505	0.3058	0.020*	
C4	0.6399 (3)	0.44345 (17)	0.43988 (16)	0.0181 (5)	
H4	0.5525	0.4619	0.4480	0.022*	
C5	0.7483 (3)	0.42455 (17)	0.51175 (16)	0.0185 (5)	
H5	0.7362	0.4295	0.5689	0.022*	
C6	0.8786 (2)	0.39750 (16)	0.50003 (15)	0.0143 (5)	
C7	0.9995 (3)	0.37848 (16)	0.56932 (15)	0.0169 (5)	
H7	0.9952	0.3826	0.6283	0.020*	
C8	1.1213 (3)	0.35443 (16)	0.55191 (15)	0.0157 (5)	
H8	1.2016	0.3415	0.5987	0.019*	
C9	1.1288 (2)	0.34858 (15)	0.46464 (15)	0.0140 (5)	
C10	1.2609 (2)	0.32358 (17)	0.44237 (16)	0.0177 (5)	
H10A	1.2913	0.3781	0.4135	0.027*	
H10B	1.2447	0.2683	0.4030	0.027*	
H10C	1.3332	0.3079	0.4961	0.027*	
C11	0.4758 (2)	0.37589 (15)	−0.01425 (15)	0.0124 (5)	
C12	0.5986 (2)	0.38162 (15)	0.05651 (15)	0.0127 (5)	
C13	0.7208 (2)	0.40884 (16)	0.03862 (15)	0.0144 (5)	
H13	0.8044	0.4129	0.0847	0.017*	
C14	0.7223 (2)	0.43073 (16)	−0.04781 (16)	0.0157 (5)	
H14	0.8076	0.4494	−0.0588	0.019*	
C15	0.6052 (2)	0.42585 (16)	−0.11591 (15)	0.0157 (5)	
H15	0.6090	0.4413	−0.1735	0.019*	
C16	0.4782 (2)	0.39768 (15)	−0.10042 (15)	0.0133 (5)	
C17	0.3494 (2)	0.39320 (16)	−0.16628 (16)	0.0164 (5)	
H17	0.3470	0.4067	−0.2253	0.020*	
C18	0.2294 (3)	0.36989 (16)	−0.14609 (16)	0.0178 (5)	
H18	0.1438	0.3684	−0.1908	0.021*	
C19	0.2312 (2)	0.34802 (16)	−0.05948 (15)	0.0152 (5)	
C20	0.1026 (2)	0.32331 (18)	−0.03312 (17)	0.0212 (5)	
H20A	0.1008	0.3598	0.0192	0.032*	
H20B	0.1025	0.2548	−0.0204	0.032*	
H20C	0.0202	0.3392	−0.0809	0.032*	
C21	0.3867 (3)	0.27543 (19)	0.24135 (16)	0.0218 (5)	
H21A	0.4864	0.2742	0.2452	0.033*	
H21B	0.3623	0.2186	0.2701	0.033*	
H21C	0.3652	0.3329	0.2704	0.033*	

### Atomic displacement parameters (Å^2^)

**Table:**

	*U*^11^	*U*^22^	*U*^33^	*U*^12^	*U*^13^	*U*^23^
Br1	0.01686 (12)	0.02067 (12)	0.01393 (12)	−0.00406 (9)	0.00458 (9)	−0.00180 (9)
Br2	0.02141 (13)	0.02021 (12)	0.02170 (14)	−0.00056 (10)	0.01060 (11)	0.00474 (10)
Zn1	0.01209 (14)	0.02082 (14)	0.00922 (14)	−0.00032 (11)	0.00298 (10)	0.00126 (11)
O1	0.0102 (8)	0.0304 (9)	0.0080 (8)	0.0014 (7)	0.0003 (6)	0.0007 (7)
O2	0.0124 (8)	0.0244 (9)	0.0078 (8)	−0.0009 (7)	−0.0005 (6)	0.0012 (7)
O3	0.0159 (9)	0.0454 (12)	0.0159 (9)	−0.0032 (9)	0.0056 (7)	0.0036 (8)
N1	0.0126 (9)	0.0126 (9)	0.0103 (9)	−0.0021 (8)	0.0024 (8)	0.0006 (7)
N2	0.0143 (10)	0.0131 (9)	0.0103 (10)	0.0007 (8)	0.0019 (8)	0.0002 (7)
C1	0.0154 (12)	0.0108 (10)	0.0119 (11)	−0.0042 (9)	0.0051 (9)	−0.0005 (8)
C2	0.0157 (12)	0.0144 (11)	0.0119 (12)	−0.0035 (9)	0.0032 (9)	0.0000 (9)
C3	0.0130 (11)	0.0203 (12)	0.0154 (12)	−0.0033 (9)	0.0011 (10)	0.0006 (9)
C4	0.0150 (12)	0.0197 (12)	0.0217 (13)	−0.0031 (10)	0.0084 (10)	−0.0042 (10)
C5	0.0270 (14)	0.0165 (12)	0.0152 (12)	−0.0053 (10)	0.0113 (11)	−0.0031 (10)
C6	0.0189 (12)	0.0124 (11)	0.0116 (11)	−0.0042 (9)	0.0043 (10)	0.0006 (9)
C7	0.0262 (13)	0.0152 (12)	0.0090 (12)	−0.0049 (10)	0.0045 (10)	−0.0006 (9)
C8	0.0197 (12)	0.0131 (11)	0.0120 (12)	−0.0016 (9)	0.0003 (10)	0.0007 (9)
C9	0.0177 (12)	0.0097 (11)	0.0144 (12)	−0.0032 (9)	0.0039 (10)	0.0010 (9)
C10	0.0158 (12)	0.0202 (12)	0.0153 (13)	0.0008 (10)	0.0013 (10)	0.0005 (10)
C11	0.0160 (12)	0.0095 (10)	0.0118 (11)	0.0033 (9)	0.0041 (9)	−0.0007 (8)
C12	0.0153 (11)	0.0120 (11)	0.0100 (11)	0.0022 (9)	0.0018 (9)	−0.0016 (8)
C13	0.0114 (11)	0.0165 (11)	0.0140 (12)	0.0022 (9)	0.0012 (9)	−0.0018 (9)
C14	0.0142 (12)	0.0155 (11)	0.0199 (13)	0.0023 (9)	0.0090 (10)	−0.0004 (9)
C15	0.0212 (13)	0.0161 (11)	0.0112 (12)	0.0023 (10)	0.0065 (10)	−0.0001 (9)
C16	0.0168 (12)	0.0108 (10)	0.0115 (11)	0.0049 (9)	0.0021 (9)	−0.0008 (9)
C17	0.0217 (13)	0.0139 (11)	0.0134 (12)	0.0052 (9)	0.0042 (10)	−0.0012 (9)
C18	0.0175 (12)	0.0186 (12)	0.0144 (12)	0.0021 (10)	−0.0009 (10)	−0.0022 (9)
C19	0.0152 (12)	0.0114 (11)	0.0175 (13)	0.0007 (9)	0.0017 (10)	−0.0005 (9)
C20	0.0145 (12)	0.0254 (13)	0.0220 (14)	−0.0006 (10)	0.0023 (11)	0.0035 (11)
C21	0.0207 (13)	0.0275 (14)	0.0160 (13)	−0.0011 (11)	0.0032 (10)	0.0022 (10)

### Geometric parameters (Å, °)

**Table:**

Br1—Zn1	2.3758 (4)	C8—H8	0.9500
Br2—Zn1	2.3637 (4)	C9—C10	1.496 (3)
Zn1—O1	1.9828 (16)	C10—H10A	0.9800
Zn1—N1	2.0431 (19)	C10—H10B	0.9800
O1—C2	1.335 (3)	C10—H10C	0.9800
O2—C12	1.341 (3)	C11—C16	1.409 (3)
O2—H2	0.8400	C11—C12	1.421 (3)
O3—C21	1.423 (3)	C12—C13	1.378 (3)
O3—H3	0.8400	C13—C14	1.411 (3)
N1—C9	1.331 (3)	C13—H13	0.9500
N1—C1	1.370 (3)	C14—C15	1.359 (3)
N2—C19	1.335 (3)	C14—H14	0.9500
N2—C11	1.368 (3)	C15—C16	1.411 (3)
N2—H2N	0.8600	C15—H15	0.9500
C1—C6	1.408 (3)	C16—C17	1.419 (3)
C1—C2	1.434 (3)	C17—C18	1.360 (3)
C2—C3	1.379 (3)	C17—H17	0.9500
C3—C4	1.409 (3)	C18—C19	1.404 (3)
C3—H3a	0.9500	C18—H18	0.9500
C4—C5	1.367 (3)	C19—C20	1.494 (3)
C4—H4	0.9500	C20—H20A	0.9800
C5—C6	1.414 (3)	C20—H20B	0.9800
C5—H5	0.9500	C20—H20C	0.9800
C6—C7	1.418 (3)	C21—H21A	0.9800
C7—C8	1.359 (3)	C21—H21B	0.9800
C7—H7	0.9500	C21—H21C	0.9800
C8—C9	1.411 (3)		
			
O1—Zn1—N1	83.77 (7)	H10A—C10—H10B	109.5
O1—Zn1—Br2	113.91 (5)	C9—C10—H10C	109.5
N1—Zn1—Br2	112.22 (5)	H10A—C10—H10C	109.5
O1—Zn1—Br1	109.87 (5)	H10B—C10—H10C	109.5
N1—Zn1—Br1	121.55 (5)	N2—C11—C16	119.6 (2)
Br2—Zn1—Br1	112.362 (14)	N2—C11—C12	119.2 (2)
C2—O1—Zn1	111.41 (13)	C16—C11—C12	121.1 (2)
C12—O2—H2	109.5	O2—C12—C13	125.7 (2)
C21—O3—H3	109.5	O2—C12—C11	116.1 (2)
C9—N1—C1	119.98 (19)	C13—C12—C11	118.2 (2)
C9—N1—Zn1	130.41 (16)	C12—C13—C14	120.4 (2)
C1—N1—Zn1	109.43 (14)	C12—C13—H13	119.8
C19—N2—C11	123.3 (2)	C14—C13—H13	119.8
C19—N2—H2N	118.3	C15—C14—C13	121.9 (2)
C11—N2—H2N	118.3	C15—C14—H14	119.0
N1—C1—C6	122.3 (2)	C13—C14—H14	119.0
N1—C1—C2	116.5 (2)	C14—C15—C16	119.5 (2)
C6—C1—C2	121.2 (2)	C14—C15—H15	120.3
O1—C2—C3	123.6 (2)	C16—C15—H15	120.3
O1—C2—C1	118.8 (2)	C11—C16—C15	118.9 (2)
C3—C2—C1	117.6 (2)	C11—C16—C17	117.1 (2)
C2—C3—C4	120.8 (2)	C15—C16—C17	124.0 (2)
C2—C3—H3a	119.6	C18—C17—C16	120.9 (2)
C4—C3—H3a	119.6	C18—C17—H17	119.5
C5—C4—C3	122.0 (2)	C16—C17—H17	119.5
C5—C4—H4	119.0	C17—C18—C19	120.4 (2)
C3—C4—H4	119.0	C17—C18—H18	119.8
C4—C5—C6	119.2 (2)	C19—C18—H18	119.8
C4—C5—H5	120.4	N2—C19—C18	118.6 (2)
C6—C5—H5	120.4	N2—C19—C20	118.6 (2)
C1—C6—C5	119.1 (2)	C18—C19—C20	122.8 (2)
C1—C6—C7	116.5 (2)	C19—C20—H20A	109.5
C5—C6—C7	124.4 (2)	C19—C20—H20B	109.5
C8—C7—C6	120.4 (2)	H20A—C20—H20B	109.5
C8—C7—H7	119.8	C19—C20—H20C	109.5
C6—C7—H7	119.8	H20A—C20—H20C	109.5
C7—C8—C9	120.3 (2)	H20B—C20—H20C	109.5
C7—C8—H8	119.9	O3—C21—H21A	109.5
C9—C8—H8	119.9	O3—C21—H21B	109.5
N1—C9—C8	120.6 (2)	H21A—C21—H21B	109.5
N1—C9—C10	117.2 (2)	O3—C21—H21C	109.5
C8—C9—C10	122.1 (2)	H21A—C21—H21C	109.5
C9—C10—H10A	109.5	H21B—C21—H21C	109.5
C9—C10—H10B	109.5		
			
N1—Zn1—O1—C2	3.23 (14)	C6—C7—C8—C9	0.3 (3)
Br2—Zn1—O1—C2	−108.35 (14)	C1—N1—C9—C8	1.4 (3)
Br1—Zn1—O1—C2	124.58 (13)	Zn1—N1—C9—C8	176.10 (15)
O1—Zn1—N1—C9	−178.0 (2)	C1—N1—C9—C10	−178.93 (19)
Br2—Zn1—N1—C9	−64.7 (2)	Zn1—N1—C9—C10	−4.3 (3)
Br1—Zn1—N1—C9	72.5 (2)	C7—C8—C9—N1	−1.1 (3)
O1—Zn1—N1—C1	−2.90 (14)	C7—C8—C9—C10	179.2 (2)
Br2—Zn1—N1—C1	110.42 (13)	C19—N2—C11—C16	−2.7 (3)
Br1—Zn1—N1—C1	−112.43 (13)	C19—N2—C11—C12	176.3 (2)
C9—N1—C1—C6	−0.9 (3)	N2—C11—C12—O2	0.2 (3)
Zn1—N1—C1—C6	−176.60 (17)	C16—C11—C12—O2	179.15 (19)
C9—N1—C1—C2	177.82 (19)	N2—C11—C12—C13	−179.13 (19)
Zn1—N1—C1—C2	2.1 (2)	C16—C11—C12—C13	−0.2 (3)
Zn1—O1—C2—C3	176.09 (18)	O2—C12—C13—C14	−179.0 (2)
Zn1—O1—C2—C1	−3.1 (2)	C11—C12—C13—C14	0.3 (3)
N1—C1—C2—O1	0.6 (3)	C12—C13—C14—C15	−0.1 (3)
C6—C1—C2—O1	179.3 (2)	C13—C14—C15—C16	−0.3 (3)
N1—C1—C2—C3	−178.6 (2)	N2—C11—C16—C15	178.7 (2)
C6—C1—C2—C3	0.1 (3)	C12—C11—C16—C15	−0.2 (3)
O1—C2—C3—C4	−179.7 (2)	N2—C11—C16—C17	1.1 (3)
C1—C2—C3—C4	−0.6 (3)	C12—C11—C16—C17	−177.8 (2)
C2—C3—C4—C5	0.4 (4)	C14—C15—C16—C11	0.4 (3)
C3—C4—C5—C6	0.3 (4)	C14—C15—C16—C17	177.9 (2)
N1—C1—C6—C5	179.2 (2)	C11—C16—C17—C18	0.8 (3)
C2—C1—C6—C5	0.6 (3)	C15—C16—C17—C18	−176.8 (2)
N1—C1—C6—C7	0.1 (3)	C16—C17—C18—C19	−1.2 (3)
C2—C1—C6—C7	−178.6 (2)	C11—N2—C19—C18	2.2 (3)
C4—C5—C6—C1	−0.7 (3)	C11—N2—C19—C20	−177.2 (2)
C4—C5—C6—C7	178.4 (2)	C17—C18—C19—N2	−0.3 (3)
C1—C6—C7—C8	0.2 (3)	C17—C18—C19—C20	179.1 (2)
C5—C6—C7—C8	−178.9 (2)		

### Hydrogen-bond geometry (Å, °)

**Table:**

*D*—H···*A*	*D*—H	H···*A*	*D*···*A*	*D*—H···*A*
O2—H2···O1	0.84	1.71	2.546 (2)	172
O3—H3···Br1^i^	0.84	2.48	3.2941 (17)	163
N2—H2n···O3	0.86	1.91	2.739 (3)	162

Symmetry codes: (i) *x*−1, *y*, *z*.
